# Enigma of Retrotransposon Biology in Mammalian Early Embryos and Embryonic Stem Cells

**DOI:** 10.1155/2018/6239245

**Published:** 2018-07-15

**Authors:** Ying Yin, Liquan Zhou, Shuiqiao Yuan

**Affiliations:** ^1^Craniofacial and Skeletal Diseases Branch, NIDCR, NIH, Bethesda, MD, USA; ^2^Family Planning Research Institute, Tongji Medical College, Huazhong University of Science and Technology, Wuhan, China

## Abstract

Retrotransposons comprise a significant fraction of mammalian genome with unclear functions. Increasing evidence shows that they are not just remnants of ancient retroviruses but play important roles in multiple biological processes. Retrotransposons are epigenetically silenced in most somatic tissues and become reactivated in early embryos. Notably, abundant retrotransposon expression in mouse embryonic stem cells (ESCs) marks transient totipotency status, while retrotransposon enrichment in human ESCs indicates naive-like status. Some retrotransposon elements retained the capacity to retrotranspose, such as LINE1, producing genetic diversity or disease. Some other retrotransposons reside in the vicinity of endogenous genes and are capable of regulating nearby genes and cell fate, possibly through providing alternative promoters, regulatory modules, or orchestrating high-order chromatin assembly. In addition, retrotransposons may mediate epigenetic memory, regulate gene expression posttranscriptionally, defend virus infection, and so on. In this review, we summarize expression patterns and regulatory functions of different retrotransposons in early embryos and ESCs, as well as document molecular mechanisms controlling retrotransposon expression and their potential functions. Further investigations on the regulatory network of retrotransposons in early embryogenesis and ESCs will provide valuable insights and a deeper understanding of retrotransposon biology. Additionally, endeavors made to unveil the roles of these mysterious elements may facilitate stem cell status conversion and manipulation of pluripotency.

## 1. Background

Approximately 40% of the mammalian genome is comprised of retrotransposons, implying their important role in organizing genomic architecture, orchestrating biological processes, and contributing to species diversity and evolution. Mammalian retrotransposons include non-LTR retrotransposons and LTR retrotransposons (also known as endogenous retroviruses, ERVs). Non-LTR retrotransposons mainly include long/short interspersed nuclear elements (LINEs and SINEs). LINE1 is the most well-studied non-LTR retrotransposon because it is active in both mouse and human, while mobilization of SINEs relies on LINE1-encoded proteins [1]. During mobilization, LINE1 is transcribed and translated and then reverse transcribed and integrated back into the genome, with a slight preference for intergenic genomic regions [2]. Differently, ERVs such as intracisternal A-particle (IAP) are active in mice, but not mobilized in human except in some pathological conditions [3]. Therefore, human ERVs are always regarded as genomic fossils of ancient retroviruses and descendants.

The most intriguing question about retrotransposon is its biological function. Basically, a retrotransposition event leads to insertional mutagenesis and may change gene structure and expression, depending on insertion position and direction. Retrotransposons may interfere with gene expression by antisense transcription or premature transcription termination. Alternatively, retrotransposons may provide new transcription start sites to change gene regulation and gene structure. In addition, retrotransposon-contained regulatory elements such as enhancers allow target genes to acquire new expression and regulatory patterns. Sometimes cytoplasmic mRNA is incorporated during retrotransposon complex assembly; then later, this gene sequence may be inserted into the genome by retrotransposition activity and create pseudogenes that might gain new functions during evolution. Moreover, retrotransposon sequences may mediate genomic rearrangement through nonallelic homologous recombination. Despite this knowledge, how retrotransposon functions remains largely unknown and controversial, while recent progress on retrotransposons opens up new insights into understanding their functional importance. Several reviews have been published regarding structures and potential functions of retrotransposons [4–7]. Here, we focus on the systems of early embryos and embryonic stem cells (ESCs) to summarize how retrotransposons are activated dynamically, discuss how these elements are regulated, and how they are involved in various biological processes like genetic innovation, cell fate change, and epigenetic memory.

## 2. Retrotransposons Are Silenced in Somatic Cell Types

Retrotransposon elements are potentially destructive to mammalian genome because of their transposition activities which may change target DNA sequences. To protect host genome from the deleterious effect in somatic tissues, retrotransposons are recognized by factors like KRAB-ZFPs and KAP1 (TRIM28) and completely silenced through DNA methylation, histone methylation/acetylation, and posttranscriptional regulation [8], except in the brains where they provide genetic variations among neurons [9]. LINE1 elements are always repressed by DNMT1-mediated DNA methylation, while ERVs are mainly inhibited through histone modification like KAP1-mediated H3K9me3. Notably, epigenetic marks crosstalk to secure consistent transposon silencing [10]. Other mechanisms have also been reported, including small interfering RNA (siRNA) pathway [11], microprocessor for miRNA biogenesis [12], RNA editing [13], and autophagy [14]. Despite these silencing mechanisms, newly evolved retrotransposons may still escape surveillance and inhibition; hence, additional repressing mechanisms may exist. Removal of repressing epigenetic marks is an important indication of several types of cancer [15]. Hence, investigations on abnormalities driven by retrotransposon activation in cancer cells will facilitate uncovering retrotransposon biology.

## 3. Retrotransposons Are Robustly Expressed in Early Embryos

Retrotransposons are activated and robustly expressed in preimplantation embryos. Although sperm and oocytes are epigenetically distinct and diversely differentiated, male and female genomes are similar in chromatin opening and gene activation after fertilization [16, 17], including retrotransposon loci [18]. Through systematic analysis of full transcriptome of repetitive elements in different stages of early mouse embryos [18], 15% to 20% of the whole transcriptome was identified as retrotransposons. Both non-LTR retrotransposons and LTR retrotransposons have the highest expression at 2-cell stage and are inhibited afterwards by the loss of active histone modifications without acquisition of repressing histone modifications. Similarly, human retrotransposons are activated from 8-cell stage and are gradually downregulated in later developmental stages, concomitantly with embryonic genome activation [19]. In early embryos, retrotransposons located in the vicinity of host genes may play a role of alternative promoters or regulatory modules, like enhancers, to activate embryonic genes [19, 20].

Retrotransposons are not only transcribed in early embryos but also have the capacity to be translated and assembled into virus-like particles. For LINE1 element, its RNA and proteins are abundant in germ cells and early embryos and are rarely found in somatic cells. However, transgenic mouse model carrying mouse or human LINE1 retrotransposition reporter showed that LINE1 integration events mainly happen in early embryos instead of germ cells [21], possibly because of significant epigenetic reprogramming and chromatin opening in the development window of preimplantation development. Likewise, ERV RNA and proteins are also present in early embryos to ensure successful embryogenesis, but their functions remain elusive and need further exploration [19, 22].

## 4. Retrotransposons Are Transiently Activated in ESCs

Although mouse and human ESCs are globally transcriptionally hyperactive, retrotransposons are mostly silent in these pluripotent cells. Here, pluripotency is based on the ability of ESCs to differentiate into all cell types of three embryonic germ layers. However, pluripotent stem cells are referred to as in naive status if they correspond to inner cell mass in preimplantation blastocyst and convert to primed status if they represent postimplantation epiblast cells; only naive pluripotent cells are able to generate chimeras. Naive and primed ESCs are distinct in their epigenetic status, such as histone modification pattern at promoters [23, 24] and enhancers [25]. Mouse and human ESCs are both derived from blastocysts but exhibit different cellular and molecular characteristics, with mouse ESCs at naive status and human ESCs at primed status based on their biological properties.

Despite global silencing of retrotransposons in mouse ESCs, interestingly, a transient small population of mouse ESCs is enriched in ERV expression [26]. This population has the capacity to contribute to both inner cell mass and trophectoderm in blastocyst and has expanded cell fate potential into both embryonic and extraembryonic cell types, differently from conventional ESCs which are pluripotent and only contribute to inner cell mass and develop into embryonic cell types. This expanded cell fate potential is called totipotency [27]. Therefore, ERV activation is regarded as a hallmark of totipotency in mice.

For human ESCs, analysis of signaling and epigenetic features identified them to be similar to mouse-derived epiblast stem cells (EpiSCs) [28] which show primed pluripotency to differentiate into specific cell lineages. Efforts have been made to fully understand fundamental properties of human naive status and how primed-to-naive transition could be facilitated [28–35]. Recently, it is reported that in human ESCs, robust ERV expression is a common and consistent feature in multiple human naive ESCs with different genetic backgrounds and derivation or culture conditions [36]. Human endogenous retrovirus subfamily H (HERVH) is one of the most important examples of retrotransposon because it is abundant in ESCs and embryos and marks pluripotency [37–40]. Additionally, ERVH abundance was also used to purify naive-like human ESC subpopulation to reveal complexity of naive-like pluripotency [41]. It is recognized that activation of human ERV is facilitated by both DNA hypomethylation and OCT4 transactivation, with ERV-encoded retroviral proteins detectable at naive status [19]. As a result, activation of retrotransposons in human ESCs is a hallmark for naive-like property and provides concordance between cell status and early developmental stage [42].

## 5. Functional Roles of Retrotransposons

Abundant retrotransposon expression in early embryos and transient ESC populations suggests possible roles of these elements during this developmental window, which is supported by the fact that cleavage-stage genes of the early embryonic transcriptional network including those driven by retrotransposons are conserved among species [43]. However, it should be kept in mind that a significant number of loci driven by retrotransposons lack orthologs among species, indicating recent retrotransposition activity without developmental functions. Loss of function assay of retrotransposons through element deletion [44] or RNA knockdown [22, 45] also shows that retrotransposon activity is essential for fertility and embryo development, with their detailed functions in host genome gradually unveiled and expanded.

### 5.1. Dynamic Balancing of Genomic Stability and Genetic Innovation

Retrotransposon mobilization during early embryogenesis causes somatic mosaicism [21], which increases genetic variability among individuals and may attribute to discrepancy of susceptibility to environmental stresses. In a mouse model of increased LINE1 expression in oocytes, although genetic diversity was increased, meiotic defects, reduced oocyte quality, and embryonic lethality were identified at the same time [46]. Additionally, abnormal activation of retrotransposons in somatic cells was found to reduce genetic instability [47, 48]. Therefore, the battle between retrotransposon mobilization and silencing mechanisms seems to be balanced between increased genetic diversity and genome stability.

### 5.2. Modulating Transcriptional and Posttranscriptional Regulatory Network

It is reported that 6–30% of mouse and human capped-RNA transcripts initiate within repetitive elements [20]. In this way, retrotransposons drive activation of developmental genes to participate in biological processes and regulate cell fate. Among retrotransposons, ERV element is highly abundant in early mouse and human embryos and provides alternative promoter or regulatory element to drive downstream gene activation in multiple loci [49]. One interesting example is a nuclear long intergenic noncoding RNA (lincRNA), LincGET, which is ERV-associated and 2-to-4-cell embryo specific [50]. Microinjection assay showed its necessity for preimplantation development, possibly through regulating gene transcription and alternative splicing. Concomitantly, ERV-enriched subpopulation of mouse/human ESCs activates a subset of early embryonic genes, differently from conventional ESCs [26, 36]. In mouse ESCs, the absence of replacement histone variant H3.3, which is important for cell fate decision in early embryos [51], leads to reduced repressing histone mark and upregulated ERV expression, causing derepression of endogenous genes in vicinity [52]. It is also reported that depletion of miR-34a in mouse ESCs leads to significantly enhanced ERV expression and acquisition of totipotency, implicating that ERV is involved in a complicated molecular network with both transcriptional and posttranscriptional regulations [53]. Recently, it is shown that several mammalian ERV elements contain DUX (mouse)/DUX4 (human) binding sites, and these sites are activated with other embryonic genes by DUX/DUX4 in mouse/human ESCs to acquire expanded developmental potency [43, 54, 55].

Other evidence indicates that retrotransposons also regulate gene expression posttranscriptionally. ERV components have been reported to interact with cellular RNAs to increase their translational activity, identified through ribosome profiling analysis [19]. SINE elements orchestrate pre-mRNA splicing [56], alter mRNA turnover when present at 3'UTR of transcripts [57], or mediate RNA–RNA interactions through base pairing of transcripts with SINE insertions [58].

### 5.3. Organizing High-Order Chromatin Structure

Retrotransposons are enriched in transcription factor-binding sites, including the binding site of an important chromatin organizer, CTCF. CTCF is the master regulator of high-order chromatin structure, and its binding sites in the genome are highly conserved among mammals [59]. CTCF has multiple functions, including chromatin loop mediator, long-range enhancer-promoter connector, insulating epigenetic spreading as chromatin barrier, and topological domain border as TAD boundary. Additionally, recent reports on the high-order chromatin structure of mammalian embryos further emphasize important chromatin organization roles of CTCF during early embryogenesis [60–63]. In a study on human ERV elements, transcription factor binding was systematically determined based on publicly available ChIP-seq datasets of nearly 100 transcription factors, and ERVs were identified to be bound by pluripotent factors, developmental regulators, hematopoietic factors, and CTCF [64]. In another study which analyzed differential CTCF binding in 6 representative mammals (including mouse and human), it was found that SINE repeats are enriched in CTCF binding to generate new CTCF-binding sites during evolution, and retrotransposition events during evolution produced expansions of CTCF binding at genome in a species-specific manner [59].

### 5.4. Epigenetic Memory of Acquired Characteristics of Inheritance

More and more evidence proves intergenerational inheritance of acquired metabolic disorders, with detailed mechanisms unclear. Recently, tsRNA was identified to mediate intergenerational inheritance through sperm [65, 66], and ERV is silenced by tsRNA posttranscriptionally [67]. This activity was observed in both early mouse embryos and mouse ESCs [65]. Therefore, crosstalk between tsRNA and ERV may contribute to germline transmission of environmental clues.

### 5.5. Inhibition of Virus Infection

Most ERVs acquire mutations and lose translation activity, except some recently active and preserved ones. HERVK is the most recently acquired human retrotransposon, which encodes an accessory protein Rec. HERVK is highly enriched in early human embryos and naive-like human ESCs. Ectopic overexpression of Rec in pluripotent cells leads to significant upregulation of interferon-induced viral restriction factor IFITM1 and increased innate antiviral responses, suggesting that HERVK also induces viral restriction pathway in early embryos [19]. Notably, it is reported that upregulation of ERV expression in cancer cells induces cytosolic sensing of double-stranded RNA and triggers interferon response to defend exogenous retroviral infection [68].

## 6. Conclusions

In mammals, retrotransposons are repressed in somatic cells and conventional ESCs to shelter genome from potential detrimental influence of retrotransposition. Retrotransposons are robustly expressed during embryonic genome activation in preimplantation embryos, with LINE1 retrotransposition activity detectable in early embryos. Totipotent subpopulation in mouse ESCs and naive-like subpopulation in human ESCs are derepressed in ERV expression, concomitantly with its expression in early embryos. Investigations on regulation of mammalian retrotransposons and related mechanisms shed light on a deeper understanding of their functions. Generally, retrotransposons are silenced through DNA methylation, histone modifications, and posttranscriptional regulation. In addition, other mechanisms exist to fine-tune retrotransposon activities, including histone variant replacement, small RNA regulation, and degradation mechanism. Although retrotransposons are potentially detrimental to genome integrity, their continuous evolution for novel functionality increases genomic diversity, provides various regulations on developmental genes, attributes to high-order chromatin assembly, potentially mediates epigenetic memory through generations, and defends exogenous viral infection. However, major functions of retrotransposons remain elusive and controversial. In the future, innovative gene editing techniques combined with high-throughput single-cell transcriptome and epigenome from multiple species will greatly facilitate understanding of how retrotransposons coevolve with host genome to optimize the survival of both and unveil the mystery of retrotransposon functions and mechanisms.
